# Invasive bony destructive orbital aspergillosis in an immunocompetent child: a case report

**DOI:** 10.1186/s12348-025-00485-7

**Published:** 2025-03-19

**Authors:** Seyed Mohsen Rafizadeh, Amir Mousavi, Mohammad Taher Rajabi, Amirhossein Aghajani, Zohreh Nozarian, Amin Zand

**Affiliations:** 1 Department of Oculo-Facial Plastic and Reconstructive Surgery, Farabi Eye Hospital, Tehran University of Medical Sciences, Tehran, Iran; 2 Department of Pathology, Farabi Eye Hospital, Tehran University of Medical Sciences, Tehran, Iran

**Keywords:** Aspergillosis, Invasive, Sino-orbital, Orbit, Bone erosion, Destruction, Immunocompetent

## Abstract

**Purpose:**

To report a case of invasive sino-orbital aspergillosis, a rare condition in a healthy child. The patient presented with orbital involvement and bone destruction, an exceedingly uncommon occurrence that mimics other invasive inflammatory or neoplastic orbital lesions.

**Case presentation:**

A 4-year-old female presented with an ill-defined, irregular, erythematous mass-like lesion measuring 8 × 10 mm on the left upper eyelid. Orbital computed tomography (CT) revealed an infiltrative soft tissue mass with bone erosions and destruction on the medial side of the frontal bone, extending toward the fronto-maxillary suture in the anterior orbit. Except for the left anterior ethmoidal sinus, the other paranasal sinuses were nearly clear. Magnetic resonance imaging (MRI) showed enhancement of the adjacent dura mater near the site of bony erosion and lesion expansion. The lesion was surgically excised, with drainage of mucopurulent discharge. Pathological examination revealed necrotizing granulomatous inflammation and fungal hyphae, with *Aspergillus fumigatus* growth confirmed by culture. The patient was diagnosed with invasive orbital aspergillosis. She was treated with intravenous and then oral voriconazole, and there was no recurrence of the disease.

**Conclusions:**

Invasive orbital aspergillosis with bone destruction of the orbital walls can occur in immunocompetent individuals, including children, without any predisposing factors. It can mimic other invasive orbital diseases, leading to delayed diagnosis and treatment, which may result in life-threatening outcomes if intracranial spread occurs. Therefore, timely orbital biopsy of the lesions is crucial.

## Introduction

Invasive aspergillosis primarily affects the nose and paranasal sinuses [1]. However, in some cases, secondary orbital involvement can worsen the prognosis due to the risk of intracranial spread [2]. Sino-orbital aspergillosis can occur even in healthy individuals, often mimicking inflammatory or neoplastic orbital lesions [3, 4]. As a result, a definitive diagnosis is typically made through a biopsy of the lesion, which reveals specific hyphal patterns and non-caseating granulomatous inflammation [1, 5]. Early diagnosis and appropriate systemic antifungal treatment are crucial to preventing intracranial expansion of the infection and reducing mortality [2].

Invasive sino-orbital aspergillosis is rare in children, and orbital involvement with bony destruction is even more uncommon [5–7]. Here, we present the case of a healthy child with invasive orbital aspergillosis that caused orbital bone destruction without clear evidence of predisposing factors.

## Case report

This study adheres to the CARE guidelines [8]. All procedures were conducted in accordance with the principles outlined in the Declaration of Helsinki. Written informed consent for the publication of the report and related images was obtained from the patient’s parents, and all patient details were de-identified. Ethical approval for case reports was not required by the institutional review board.

A 4-year-old female presented to the oculoplastic clinic at Farabi Eye Hospital, Tehran, Iran, with a one-month history of an erythematous mass-like lesion on the medial side of her left upper eyelid, which had progressively enlarged over the past week. The patient had an unremarkable family, systemic, and ocular history. The parents reported no history of immunodeficiency or use of immunosuppressive medications.

**Fig. 1:**
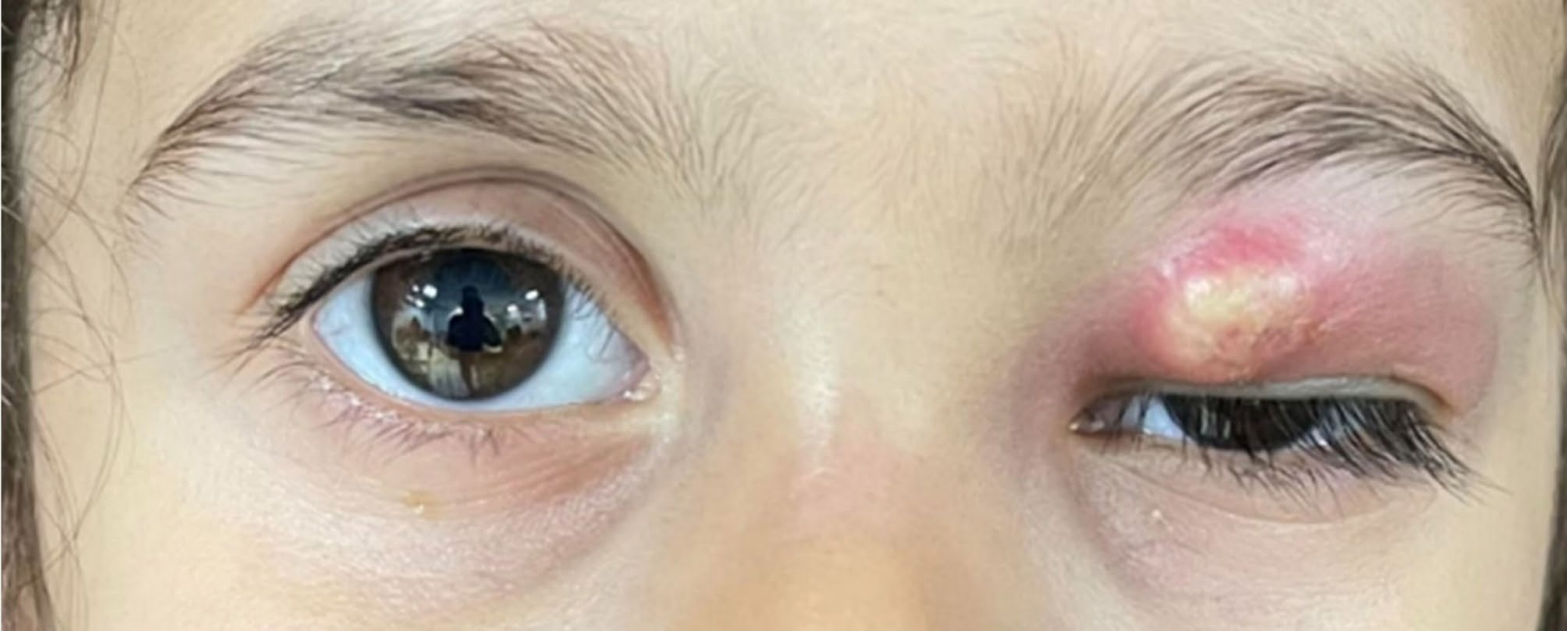
A yellowish, mass-like lesion (8 × 10 mm) located on the medial side of the left upper eyelid, accompanied by diffuse swelling and erythema

Upon examination, the patient’s best-corrected visual acuity, assessed using Allen pictures, was 20/20 in both eyes, with normal pupillary reactions and no relative afferent pupillary defect. The examination revealed an ill-defined, lobulated, and irregular erythematous mass-like lesion measuring 8 × 10 mm over the left upper eyelid (Fig. 1). Palpation revealed a semi-mobile lesion with soft components and mild tenderness. No proptosis was observed, and the orbit appeared normal without resistance to retropulsion. Mildly restricted eye movements (-1 scale) in supraduction and adduction were detected in the affected eye. Slit-lamp examination revealed no conjunctival chemosis or ciliary injection in either eye. Dilated fundus examinations were unremarkable.

Laboratory investigations showed a white blood cell count of 9.2 × 10^3^/mcL with 55% neutrophils and 5% eosinophils, an erythrocyte sedimentation rate (ESR) of 20, and a qualitative C-reactive protein of 1+. Blood cultures were negative for microbial growth.

**Fig. 2:**
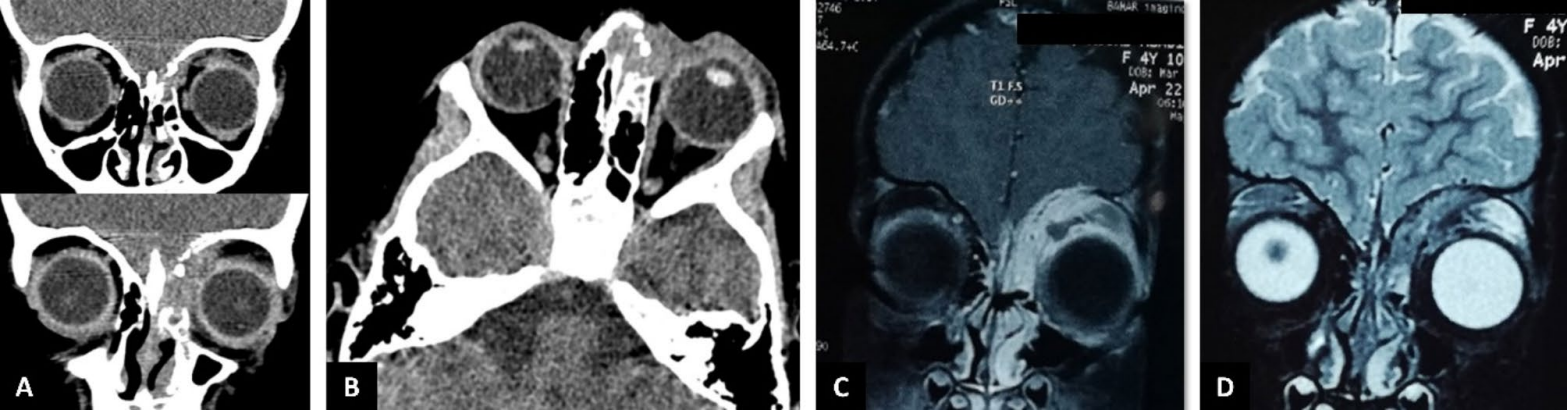
Orbital computed tomography (CT) and magnetic resonance imaging (MRI) scans. **A**: Coronal CT view showing an infiltrative, extraconal soft tissue mass in the supramedial aspect of the anterior orbit, with bone erosions and destruction in the medial frontal bone extending toward the fronto-maxillary bone suture on the left side. Maxillary sinuses were clear. **B**: Axial CT view demonstrating fullness of the left anterior ethmoidal sinus. **C**: Coronal T1-weighted MRI with gadolinium contrast revealing enhancement of the lesion and adjacent dura mater, without obvious intracranial spread. **D**: Coronal T2-weighted MRI showing the lesion to be iso- to hypointense compared to the extraocular muscles

Orbital computed tomography (CT) revealed an infiltrative soft tissue mass with bone erosions and destruction on the medial side of the frontal bone, extending toward the fronto-maxillary suture in the anterior orbit (Fig. 2A). All paranasal sinuses were clear except for mucosal thickening and fullness in the left anterior ethmoidal sinus (Fig. 2A-B). Orbital and brain magnetic resonance imaging (MRI) with contrast showed enhancement of the lesion and its adjacent dura mater in T1-weighted images, and the lesion appeared iso- to hypointense relative to the extraocular muscles in T2-weighted images (Fig. 2C-D).

**Fig. 3:**
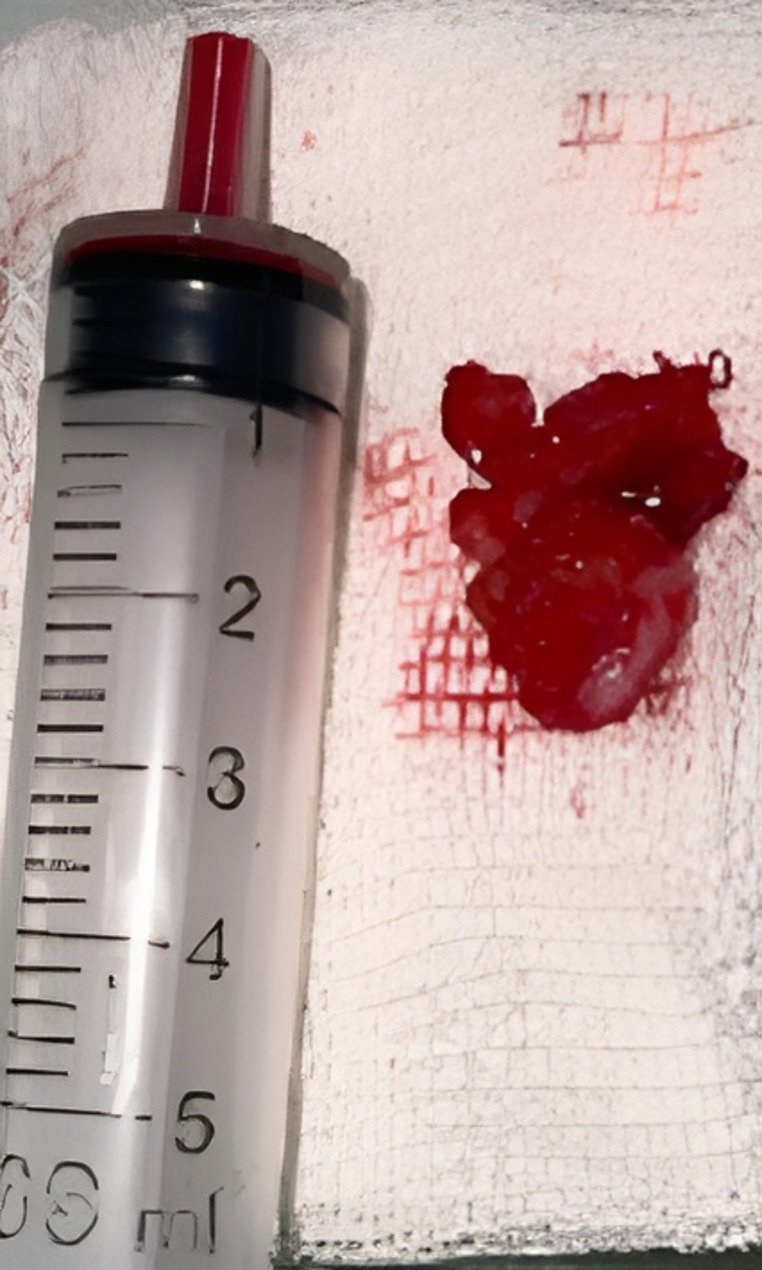
The excised mass-like lesion, located extraconally in the supramedial aspect of the anterior orbit

**Fig. 4:**
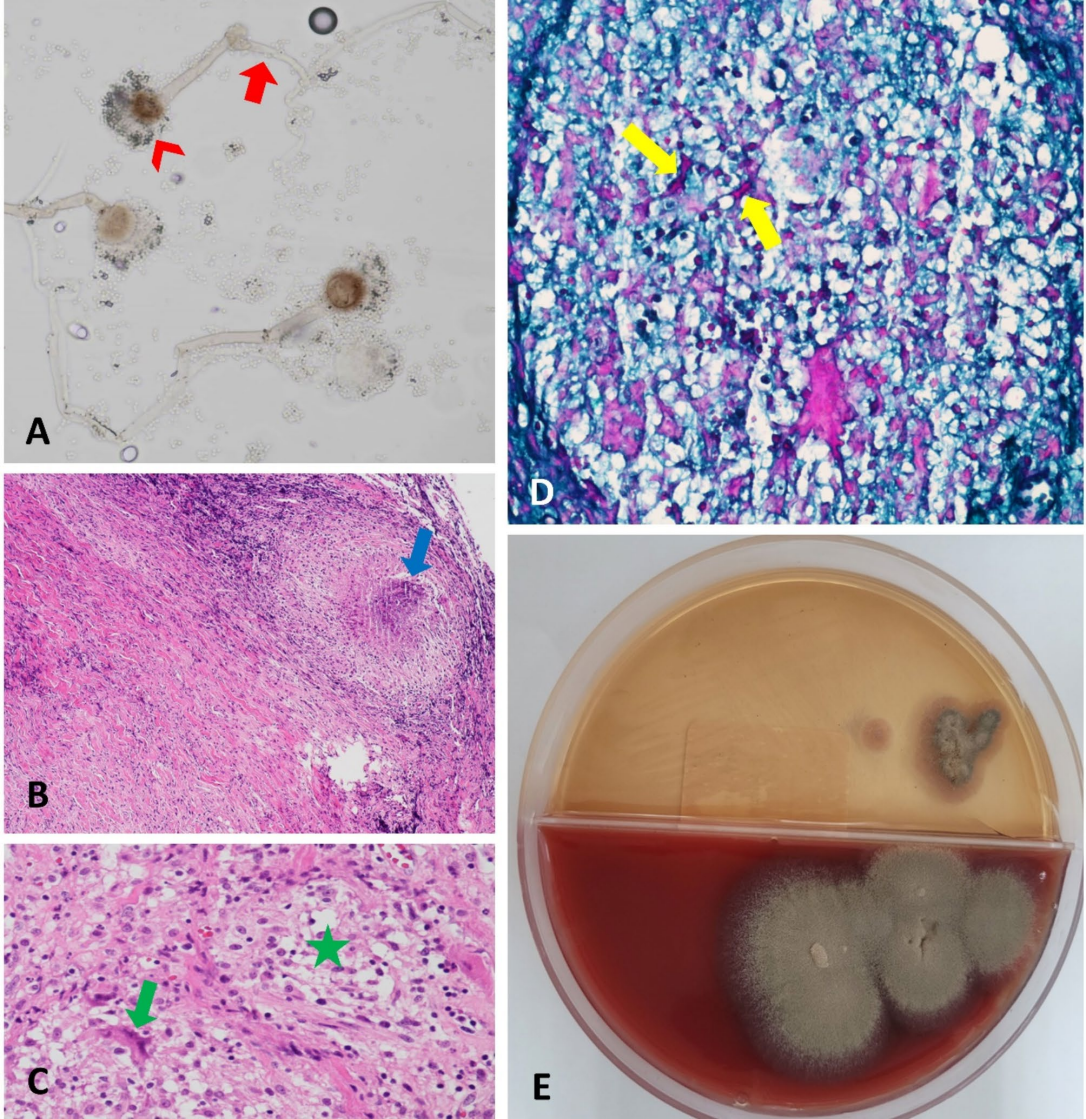
**A**: Slide culture from the fungal colony showing hyphae of uniform size with dichotomous branching at approximately 45 degrees (arrow) and specific conidia (arrowhead). **B**: Microscopic examination of fibro-connective tissue revealing infiltration of a necrotizing granulomatous lesion (arrow) (Hematoxylin and Eosin [H&E] staining; ×40 magnification). **C**: A granulomatous reaction, with aggregation of histiocytes (star) and multinucleated giant cell (arrow), was observed (H&E staining; ×400 magnification). **D**: Fungal hyphae (arrows) were visualized (Periodic acid-Schiff [PAS] staining; ×400 magnification). **E**: Growth of *Aspergillus fumigatus* was detected on Chocolate blood agar and Sabouraud agar

**Fig. 5:**
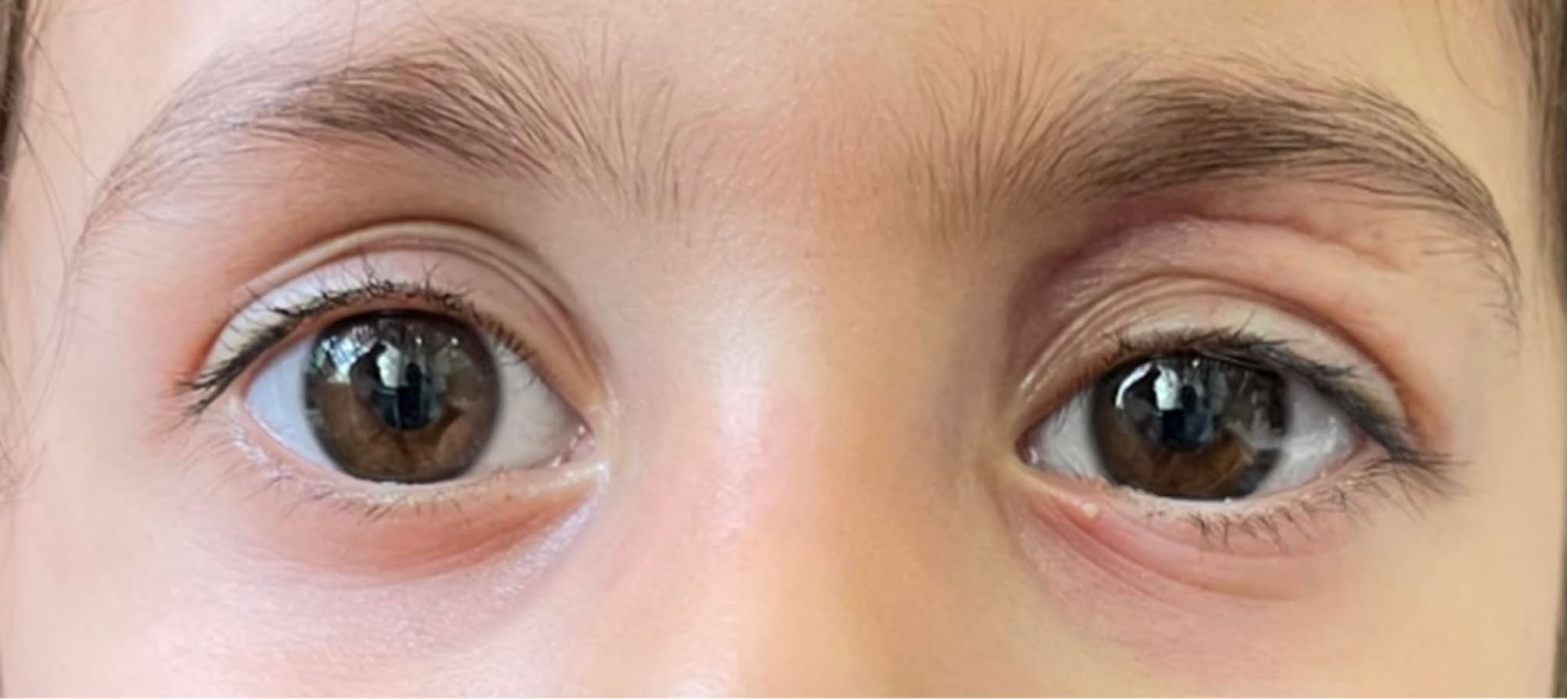
Nearly complete healing observed two weeks after surgical excision and initiation of systemic voriconazole treatment

Given the progressively enlarging lesion with bony destruction, the patient was scheduled for surgical excision to rule out neoplastic orbital lesions. An anterior orbitotomy via a trans-crease incision was performed, and components of the soft mass-like lesion were removed (Fig. 3), along with approximately 2 cc of mucopurulent discharge. Pathological examination of the discharge revealed fungal hyphae of uniform size (Fig. 4A). Further pathological evaluation of the excised soft tissue revealed necrotizing granulomatous inflammation (Fig. 4B), characterized by the aggregation of histiocytes and multinucleated giant cells (Fig. 4C). Periodic acid-Schiff (PAS) staining showed the presence of fungal hyphae (Fig. 4D). A culture on Sabouraud’s agar confirmed the growth of *Aspergillus fumigatus* (Fig. 4E). The patient was diagnosed with invasive orbital aspergillosis without obvious predisposing factors. To rule out hematogenous dissemination of the fungus from distant organs such as the lungs and liver, abdominal ultrasonography and chest X-ray were performed, both of which were unremarkable. The patient was admitted for treatment with intravenous voriconazole at 6 mg/kg/day as a loading dose for two days, followed by 4 mg/kg/day for one week, after consultation with the infectious disease service. After one week, there was significant improvement in lid swelling and erythema, and the patient was discharged with oral voriconazole (5 mg/Kg twice daily) for one month [9]. A follow-up visit one week after discharge (day 14) revealed a remarkable decrease in swelling (Fig. 5). No recurrence of signs or symptoms was observed in subsequent follow-ups.

## Discussion

Aspergillus species, commonly found in soil and decaying vegetation, are the most frequent cause of fungal infections in humans [2, 10]. Aspergillosis typically presents as an infection of the paranasal sinuses, usually in non-invasive forms such as allergic sinusitis or aspergilloma, both of which generally have a good prognosis [11]. However, in some cases, especially among immunosuppressed individuals, invasive sino-orbital aspergillosis can occur. This condition results from the extension of a primary sinonasal infection to other paranasal structures, including the orbital cavity, and can lead to life-threatening outcomes if there is intracranial spread [12]. Certain environmental factors, such as tropical climates that favor fungal growth, may also contribute to the occurrence of invasive sino-orbital aspergillosis in otherwise healthy individuals [5, 13].

The average age of patients with invasive sino-orbital aspergillosis ranges between 35 and 42 years, making our patient, a 4-year-old child, an unusual case [1, 5, 13]. Here, we report an atypical presentation of invasive orbital aspergillosis in a healthy child, characterized by significant bony destruction of the orbital walls, a rare manifestation [5, 14]. Pushker et al. reported that three out of 15 immunocompetent patients with invasive orbital aspergillosis exhibited signs of bony destruction [5]. While the disease can sometimes occur due to hematogenous dissemination from other organs such as the lungs or liver [15, 16], no definitive source of intraorbital fungal involvement was identified in our case.

Pushker et al. found that the most common clinical presentations of invasive orbital aspergillosis were proptosis, followed by limitations in extraocular movements [5]. Similarly, our patient presented with limitations in supraduction and adduction of the affected eye.

The disease does not have specific radiologic features. However, orbital CT scans in these cases typically reveal an ill-defined, infiltrative, and heterogeneous orbital mass, maybe accompanied by bone changes such as erosion or remodeling, similar to what was observed in our case [17, 18]. On MRI, these lesions appear isointense to extraocular muscles on T1-weighted images and hypointense on T2-weighted images, with heterogeneous enhancement of active lesions. MRI with contrast is also essential to rule out intracranial fungal spread [5, 19]. Although our patient’s MRI showed dural enhancement, no clear signs of intracranial spread were detected.

Systemic workup, including laboratory testing such as evaluation for eosinophilia, is generally not helpful in diagnosing the disease [5]. In our case, laboratory tests, including complete blood count, ESR, and CRP, were unremarkable.

For a definitive diagnosis, an orbital biopsy (incisional, excisional, or by fine-needle aspiration) is necessary [1, 20]. Pathological examination can identify Aspergillus hyphae by their characteristic uniform size and dichotomous branching at approximately 45 degrees [5]. Additionally, non-caseating multinucleate giant cell granulomas and eosinophilic infiltration can be observed [5, 21]. In our case, the orbital biopsy revealed specific Aspergillus hyphae along with granulomatous inflammation. Culture results confirmed the growth of *Aspergillus fumigatus*, the most commonly reported species among immunocompetent individuals [14]. Some studies suggest that PCR-based DNA analysis can be useful for accurate identification of the fungal species [22].

These patients should be treated with systemic antifungal drugs, such as intravenous amphotericin B or voriconazole [23]. Studies have shown that voriconazole has better intracranial penetration and is more tolerable than amphotericin B [12, 24]. It is recommended to continue treatment with oral antifungal drugs, such as itraconazole or voriconazole, for at least one month [5]. Considering the nephrotoxic side effects of intravenous amphotericin B, our patient, a 4-year-old girl, was treated with voriconazole. Surgical debridement of all infected tissues is also recommended due to the angioinvasive nature of the fungus, which causes necrosis through infarction and direct invasion [1, 21, 25]. Debulking of orbital lesions may improve the response to systemic antifungal treatment by enhancing drug penetration [1].

The combination of surgical debridement and systemic antifungal treatment leads to survival without recurrence in most cases [5, 26]. Pushker et al. reported that only one of 15 patients with invasive orbital aspergillosis experienced disease progression due to intracranial spread, while the others survived without recurrence [5].

In conclusion, orbital aspergillosis can occur even in immunocompetent patients with bony invasion and destruction, without any predisposing factors, and may mimic other invasive inflammatory or neoplastic orbital diseases, leading to delayed diagnosis. Therefore, performing an orbital biopsy from the lesions is crucial for early diagnosis and treatment, which can prevent the intracranial spread of the fungus and its life-threatening consequences.

## Data Availability

No datasets were generated or analysed during the current study.
